# Evaluation of Dentin Defect Formation during Retreatment with Hand and Rotary Instruments: A Micro-CT Study

**DOI:** 10.1155/2017/4868603

**Published:** 2017-05-24

**Authors:** Ayca Yilmaz, Dilek Helvacioglu-Yigit, Cansu Gur, Handan Ersev, Gullu Kiziltas Sendur, Egemen Avcu, Canan Baydemir, Paul Vincent Abbott

**Affiliations:** ^1^Department of Endodontics, Faculty of Dentistry, Istanbul University, Istanbul, Turkey; ^2^Department of Endodontics, Faculty of Dentistry, Kocaeli University, Kocaeli, Turkey; ^3^Department of Mechatronics Engineering, Faculty of Engineering and Natural Sciences, Sabanci University, Istanbul, Turkey; ^4^Department of Machine and Metal Technologies, Ford Otosan İhsaniye Automotive Vocational School, Kocaeli University, Kocaeli, Turkey; ^5^Department of Biostatistics, Kocaeli University, Kocaeli, Turkey; ^6^School of Dentistry, University of Western Australia, Perth, WA, Australia

## Abstract

The purpose of this study was to compare the incidence and longitudinal propagation of dentin defects after gutta-percha removal with hand and rotary instruments using microcomputed tomography. Twenty mandibular incisors were prepared using the balanced-force technique and scanned in a 19.9 *μ*m resolution. Following filling with the lateral compaction technique, gutta-percha was removed with ProTaper Universal Retreatment (PTUR) or hand instruments. After rescanning, a total of 24,120 cross-sectional images were analyzed. The numbers, types, and longitudinal length changes of defects were recorded. Defects were observed in 36.90% of the cross sections. A total of 73 defects were comprised of 87.67% craze lines, 2.73% partial cracks, and 9.58% fractures. No significant difference in terms of new defect formation was detected between the retreatment groups. The apical and middle portions of the roots had more dentin defects than the coronal portions. Defects in three roots of the PTUR instrument group increased in length. Under the conditions of this in vitro study, gutta-percha removal seemed to not increase the incidence of dentin defect formation, but the longitudinal defect propagation finding suggests possible cumulative dentinal damage due to additional endodontic procedures. Hand and rotary instrumentation techniques caused similar dentin defect formation during root canal retreatment.

## 1. Introduction

Vertical root fractures (VRFs) are associated with a poor prognosis for the affected tooth [1] and should therefore be prevented. Local stress concentrations have been proposed as the basis of VRFs [2]. Dentin defects, which can be created by endodontic procedures [3, 4], may act as stress concentration areas [2], propagate from repeated stresses generated as a result of further endodontic and restorative procedures [2, 5], and ultimately develop into a VRF. To avoid VRFs, safer instruments and techniques should be sought and preferred.

Several previous studies have shown that the mechanical preparation of root canals may cause dentin defect formation [3, 4]. However, only a few studies have investigated the effect of root canal retreatment procedures on the extent of dentin damage. In these studies, direct observation by optical microscopy at magnifications varying from ×12 to ×40 was used to assess the occurrence of defects in horizontal root sections obtained at various mm from the apex by using a low-speed saw [5–9] and the defect initiation and propagation on the apical root surfaces only [10, 11]. A more recent methodology, microcomputed tomographic (micro-CT) imaging, allows for nondestructive volumetric quantitative and qualitative assessments with accurate comparisons of defects in the same specimen before and after endodontic procedures. This technique has been used only to evaluate the incidence of dentin defects created by various instrumentation [12–15] and filling techniques [16]. However, to the authors' knowledge, there is no reported study regarding the effect of retreatment procedures on dentin defect formation and the longitudinal propagation of preexisting defects analyzed using micro-CT imaging. Therefore, the aim of this study was to compare the incidence and longitudinal length of dentin defects before and after the removal of gutta-percha (GP) with hand or rotary instruments using a micro-CT analysis. The null hypothesis was that there would be no difference between these two techniques.

## 2. Materials and Methods

### 2.1. Sample Size Calculation

The sample size for this study was calculated after the effect size estimation of the percentage of the dentinal defects promoted by obturation (6.7%) and retreatment procedure performed with H-files (50%) as reported by Topçuoğlu et al. [7]. An alpha-type error of 0.05 and power beta of 0.95 were also specified. Based on these parameters, eight samples were indicated as the minimum ideal size required for observing this same effect (G^*∗*^Power; Heinrich-Heine-Universität Düsseldorf, Düsseldorf, Germany).

### 2.2. Selection of Teeth

This study was approved by the Ethics Committee of Kocaeli University (Protocol number 2015/171 KOU KAEK).

Intact human mandibular central incisors with mature apices extracted for reasons unrelated to the current study were obtained from the collections of the Department of Oral and Maxillofacial Surgery, Faculty of Dentistry, Kocaeli University. Teeth with previous endodontic treatment and/or restoration, root caries, root resorption, occlusal wear, and open apices were discarded.

Digital radiographs from both buccolingual and mesiodistal directions were taken for each tooth to verify a single, patent, and straight (<5°) root canal. Teeth with pulp stones, calcified pulp spaces and root canals, and hypercementosis were excluded.

The coronal sections of the teeth were removed using a low-speed saw (Isomet 1000; Buehler, IL, USA) with water cooling to obtain standard root lengths of 12 mm and flat reference surfaces. The canal widths near the apices of all roots were compatible with a size 10 K file (Mani Inc., Tochigi-Ken, Japan).

The roots were inspected using a stereomicroscope (Leica MZ75, Leica Imaging Systems Ltd., Cambridge, UK) under ×12 magnification to exclude roots with anatomical irregularities, external defects, and cracks and to verify the presence of a single apical foramen that did not deviate from the apex. Finally, 20 teeth were selected and stored in distilled water until use.

### 2.3. Preparation and Filling of the Root Canals

The roots were embedded in acrylic resin as described previously [17] such that the resin surface was even with the most coronal flat surface of the root. The periodontal ligament was simulated [5] with a polyether impression material (Impregum Garant Soft, 3M ESPE, Seefeld, Germany). The exposed apical 3 mm of the roots were immersed in distilled water to avoid dehydration until the first and second scans were acquired, apart from the duration of the root canal filling procedure [18] and setting of the cement.

The working length (WL) was determined by inserting a size 10 K file (Mani Inc.) into each root canal until the file tip was just visible at the apical foramen. Then, 1 mm was subtracted from the measured length.

The root canals were prepared with NiTiFlex K-files (Dentsply Maillefer, Ballaigues, Switzerland) using the balanced-force technique described by Roane et al. [19]. The canals were instrumented to a master apical size of 35 and step-back technique was performed by using file sizes 40 to 50.

Irrigation was performed with 2 mL of 1% sodium hypochlorite (NaOCl) by means of a syringe and with a 27-gauge NaviTip irrigation needle (Ultradent, South Jordan, UT, USA) between each file during instrumentation. For the final irrigation, 5 mL of 17% ethylenediaminetetraacetic acid solution, 5 mL of 1% NaOCl, and 5 mL of distilled water were used. The canals were dried with paper points.

The resin cylinder containing the root was positioned coronoapically onto the designated area of the computer-controlled turntable of the high-resolution micro-CT system (SkyScan 1172, Bruker, Kontich, Belgium) and the turntable was adjusted so that the X-ray beam was perpendicular to the resin cylinder. The X-ray tube was operated at 80 kV and 128 mA with Al and Cu filters. Samples were scanned in a 360° rotation with a rotation step of 0.5°. The image pixel size was 19.9 *μ*m. Two-dimensional (2D) images were reconstructed using NRecon software (SkyScan, Bruker, Kontich, Belgium).

Master GP cones of size 35 and with a 0.02 mm/mm taper (Dentsply Maillefer) were coated with AH26 silver-free cement (Dentsply, DeTrey, Konstanz, Germany) and inserted to the WL. A finger NiTi spreader (Dentsply Maillefer) and additional GP were used for lateral compaction. The force exerted by the spreader was controlled with a household digital scale so as to not exceed 2 kg. The excess GP was seared off and the filling was vertically compacted. The access cavities were sealed with a temporary filling material (Cavit-G, 3M ESPE) and the teeth were stored at 37°C in 100% humidity for 1 month to allow complete setting of the cement.

### 2.4. Root Canal Retreatment Techniques

The teeth were divided randomly into two groups (*n* = 10) and retreated with the following technique.

*Group 1*. ProTaper Universal Retreatment (PTU-R) instruments (Dentsply Maillefer) were used in a crown-down manner with a torque-controlled electric motor (Silver RECIPROC motor, VDW, Munich, Germany) at the recommended settings. According to the manufacturer's instructions, the D1, D2, and D3 instruments were used in a brushing action with lateral pressing movements to remove the coronal, middle, and apical thirds of the root canal filling materials, respectively. All three instruments were used at 500 rpm and 2.5 Ncm. The final apical preparation was then performed by using F2, F3, and F4 PTU (Dentsply Maillefer) instruments. 

*Group 2.* The coronal portion of the root canal filling was removed using size 2 Gates-Glidden burs (Mani Inc.) and, subsequently, a size 1 bur at 1500 rpm. Hedström files (Mani Inc., sizes 30–15) were used in a circumferential, quarter-turn, push-pull filing motion to remove the filling material until the WL was achieved. Once the WL had been reached, apical preparation was performed with files up to size 40. 

The canals were irrigated with 1 mL of 1% NaOCl at each instrument change. The removal of the root canal filling was judged to be complete when the WL was reached and no filling material was observed on the final instrument. Each instrument set was used once. All canals were finally irrigated with 1 mL of 1% NaOCl and dried with paper points. A single experienced operator performed all endodontic procedures.

The teeth were repositioned on the turntable of the micro-CT system and rescanned using the aforementioned parameters.

### 2.5. Dentin Defect Evaluation

The image stacks of the roots after initial instrumentation and removal of the GP were coregistered using computer software (3DSlicer, https://www.slicer.org/).

The dentin defects were classified as follows: a “fracture” was defined as a line extending from the root canal space to the outer surface of the root [5]; a “craze line” was defined as a line extending from the outer surface into the dentin, but not reaching the canal lumen [5]; and a “partial crack” was defined as a line extending from the canal wall into the dentin without reaching the outer surface of the root [20].

Cross-sectional images (*n* = 24,120) of the apical, middle, and coronal root portions (each 4 mm in length and comprising 201 images) were screened and inspected for dentin defects by two precalibrated examiners blinded to the group allocations.

Image analyses were repeated twice at 2-week intervals to validate the screening process. In the case of disagreement, the image was examined together until consensus was reached.

First, images of the roots after GP removal were analyzed, and the number and type of defects in each cross section were recorded. Moreover, the sections presenting the start (*s*) and finish (*f*) of the defect were recorded, and the longitudinal length of the defect was calculated with the following formula: length of the defect = 19.9 *μ*m × (*f* − *s* + 1).

Then, the first set of scans was examined to verify the changes. Recordings and calculations were performed in the same way. The length changes of the defects were calculated by subtracting the second values from the first values.

### 2.6. Statistical Analysis

All statistical analyses were performed using IBM SPSS for Windows version 20.0 (SPSS, Chicago, IL, USA). Kolmogorov-Smirnov tests were used to determine the normality of data distributions. Continuous variables are expressed as the median (25th percentile to 75th percentile), and categorical variables are expressed as counts (percentage).

Comparisons of continuous paired variables were performed using Wilcoxon's *t* test, and comparisons of continuous variables between the groups were performed using the Mann–Whitney *U* test. Comparisons of categorical variables between the groups were performed using Fisher's exact test and the Monte Carlo chi-squared test. A two-sided *P* value < 0.05 was considered statistically significant.

## 3. Results

Dentin defects were observed in 4451 (36.90%) of the cross sections from both groups. Among the total of 73 defects observed following GP removal, 64 (87.67%) were craze lines, 2 (2.73%) were partial cracks, and 7 (9.58%) were fractures. Dentin defects in only three teeth in Group 1 increased in length. New defect formation was detected in only one tooth from each group (Table 1). In both groups, the apical and middle portions of the roots contained more dentin defects than the coronal portions (Table 2). There was no statistically significant difference between the groups in terms of an increased number of dentin defects (*P* > 0.05).

## 4. Discussion

The micro-CT imaging used in the current study has many advantages over sectioning methods, which have been used in most previous studies investigating dentin damage caused by various endodontic procedures [7, 9, 20–23]. Advantages of the micro-CT technique are that it is an accurate and a nondestructive approach permitting examination of the same sample before and after the experimental procedure and allowing a large number of sections to be analyzed. This technology also enables visualization of the precise location of dentin defects throughout the root and observation of their propagation after the experimental procedure. Each specimen acts as its own control, which improves the internal validity of the experiment [12]. However, this technique also poses some disadvantages, such as being a tedious, time-consuming, and, in turn, expensive task, all of which restrict the experimental procedures. Thus, the lack of the baseline micro-CT scanning of the specimens might be a limitation of the current study. However, 87.67% of the dentinal defects observed were craze lines. An external evaluation of the roots before the experimental procedures should have revealed any preexisting defects. Even if they did not, while micro-CT imaging has a much higher definition than stereomicroscopy [12], the main focus was to compare the effects of two GP removal techniques on the incidence of dentin defect formation, and the statistical comparisons were based on the meticulous evaluation of a large number (24,120) of cross-sectional slices, which were examined twice by two examiners.

Many previous studies have reported that initial root canal preparation with hand files using the balanced-force technique did not induce dentin defect formation [3, 4, 24, 25]. This has been attributed to the less aggressive movements of the hand files in the canal in comparison with NiTi rotary files [18], which require a continuous rotational movement to accomplish root canal preparation and therefore can generate a greater number of defects [24, 25]. Moreover, the counterclockwise movement performed in the balanced-force technique has been reported to reduce the penetration depth and torque [21]. In the current study, more flexible NiTi hand files were used in the initial preparation. The flexibility of an instrument was shown to have a positive impact on dentin damage formation [22]. As the primary objective of the present study was to evaluate the effect of GP removal on defect formation, the balanced-force technique performed with NiTi hand files was chosen for initial canal preparation to more precisely detect the incidence of defects generated during the subsequent retreatment procedure.

When the mechanical properties of specimens are investigated, measures taken to handle the specimens are of utmost importance in all phases of the experimental procedures. The micro-CT technology used in this study prevented mechanical damage and tissue loss from sectioning and allowed inspection of the entire specimen. High concentrations of NaOCl have been reported to significantly decrease the elastic modulus and flexural strength of dentin when used as an endodontic irrigation solution [26]. NaOCl at a 1% concentration was used in the current study to minimize the chemical insult and limit changes in the mechanical properties of the dentin during the experimental procedures. Regarding the storage conditions of the specimens, the heat generated during the polymerization of the acrylic resin utilized to embed the specimens and scanning under dry conditions may have caused spontaneous cracks in the dentin [27, 28]. This might explain the occurrence of dentin defects in 36.9% of the assessed slices after the first scanning. However, the very low incidences of new defect formation and the propagation of preexisting cracks detected in the second scanning suggest that the scanning conditions did not cause a deleterious effect on the specimens. Furthermore, cautious measures were taken to keep the roots hydrated throughout the experimental procedures.

Mandibular central incisors were chosen for this study as their roots are narrower mesiodistally, they have lesser dentin thicknesses, and their root canals have oval cross sections, all of which make these teeth most susceptible to vertical root fracture [2]. Another reason for choosing these teeth was that teeth with a single canal do not exhibit extreme variations in internal and external morphology, which enables the observation of longitudinal propagation of preexisting defects without interference from anatomical features.

It has been reported that the root canal filling technique may affect the occurrence of dentinal defects [8, 20]. Lateral compaction of GP was performed in the current study. This technique was chosen as it is widely used and one of the preferred filling techniques, but it is also an active technique that applies pressure. In comparison with passive techniques, filling techniques associated with pressure were shown to create more dentinal defects [8, 20]. It has also been stated that forces generated with spreaders may produce a wedging effect [29] and result in apical dentin defect generation [9]. Furthermore, the apical third generally has a greater amount of remaining root canal filling material [30] and additional instrumentation may be performed to reduce the amount of remaining material [31]. Additional instrumentation may lead to a greater reduction in root structure where the dentin is already thin and, thus, may create dentin defects [5]. These factors might explain the new defect occurrence at the apical level in each group, although evaluating the effect of the lateral compaction technique on the formation of defects was not an objective of the current study.

In the present study, two fractures at the apical level propagated in the coronal direction, and a fracture at the middle level propagated in both the apical and coronal directions in the PTU-R group where additional instrumentation was performed with PTU instruments up to a taper of 0.06. Although this finding did not present statistical significance, it suggests that a more tapered preparation might lead to further damage to the root dentin, as no longitudinal propagation was observed in the hand file retreatment group. Previously, more tapered instruments were shown to generate increased crack formation [22, 25]. Another explanation of the fracture propagations might be the high-stress areas in the apical third and between the apical and middle thirds, supposedly as a result of stresses transmitted via GP in these areas during lateral compaction [2].

In some studies investigating the influence of retreatment procedures on the incidence of dentin defects, it was concluded that, in most cases, GP removal creates more dentin defects than the initial mechanical preparation [5, 7–10]. It seems reasonable that performing additional treatment during the GP removal procedure would increase the incidence of dentin defects, as Shemesh et al. [6] stated. However, in the present study, a significant impact was not detected from GP removal. This difference might be the result of the methodology used herein and the relatively easy removal of laterally condensed filling material, probably because of its poor adaptation to the root canal wall [8, 32]. The lack of micro-CT scanning of the specimens before commencing the retreatment procedure in the present study might be a drawback; however, similar to our findings, Üstün et al. [9] found that the amount of dentin defects following a retreatment procedure with PTU-R files was similar to that after the initial preparation of the root canal with hand files using the balanced-force technique. In agreement with the current results, Capar et al. [8] found that the removal of laterally condensed GP filling material with PTU-R files had no effect on defect formation. Shemesh et al. [6] showed that both PTU-R and hand files created dentin defects but found no difference between the two techniques for GP removal in roots filled with the lateral compaction technique, in agreement with the current study. Thus, the null hypothesis was accepted.

**Figure 1:**
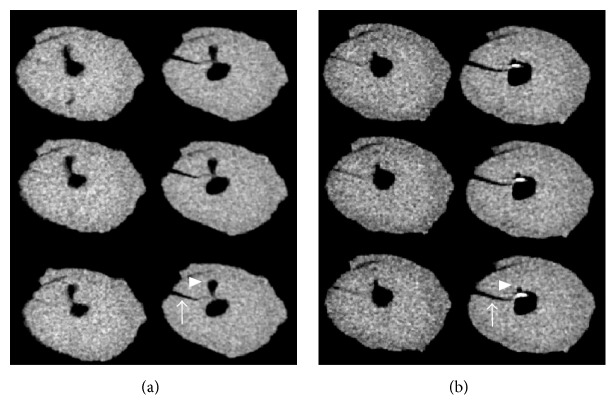
Two sets of contiguous micro-CT images of the same root are shown ((a) after initial instrumentation; (b) after the removal of gutta-percha). (a) Craze line formation (arrow) following gutta-percha removal; (b) a craze line developing into a fracture (arrow) following gutta-percha removal and lateral canal (arrowhead). Note the remaining filling material.

During the evaluation of contiguous slices, a defect was recorded as a fracture if a defect in any slice was classified as a fracture. Extensions of the fractures manifested themselves as craze lines on contiguous slices before the removal of GP. After removal, as the defect propagated longitudinally, the preexisting craze lines also propagated horizontally and became fractures, as can be seen in Figure 1. This finding could confirm that defects can act as stress concentration areas [2] and VRF, a longitudinally oriented fracture of the root [33], may develop following further endodontic and restorative procedures [5].

Onnink et al. [34] observed that a dentin defect contained within the dentin in one section could communicate with the root canal in a contiguous section. Landrigan et al. [35] used contrast-enhanced micro-CT imaging to determine the presence, morphology, and spatial locations of dentin cracks in machined, partially fractured elephant dentin specimens and in whole human molars with visual evidence of external cracks following extraction. In contrast to previous studies evaluating the effects of retreatment procedures with stereomicroscopy [10, 11], the micro-CT method used in the present study allowed monitoring of the defect extents to different levels of the root and the propagation of preexisting defects.

With a synchrotron light-based micro-CT system, Pop et al. [36] assessed the 420 most apical contiguous horizontal sections of each root and detected significant increases in the numbers and lengths of dentin defects following mechanical preparation. It was beyond the scope of the present study to determine the extent of the horizontal propagation of a preexisting defect in each slice, but further studies focusing on this issue together with longitudinal propagation would shed more light on the influence of endodontic procedures on dentin integrity. Although very small numbers of new dentin defect formations and propagations of preexisting defects following endodontic procedures were observed in the current in vitro investigation, this could be of clinical importance, while stresses created in the root at the time of treatment may manifest themselves as fractures at a later date [1] following restoration [5] and occlusal forces [37]. A VRF should be considered to be a result of a gradual diminution of the root structure rather than an instant phenomenon [33], and cumulative stresses generated by each treatment step render teeth more prone to VRFs [6, 33].

## 5. Conclusions

In conclusion, within the limitations of this in vitro study, the removal of GP seems to not increase the incidence of dentin defect formation, but the finding of the longitudinal propagation of defects suggests possible cumulative damage to the dentin due to additional endodontic procedures. Hand and rotary instrumentation techniques caused similar dentin defect formation during root canal retreatment. Micro-CT technique proved to be a useful tool in evaluating longitudinal propagation of dentinal defect.

## Figures and Tables

**Table 1:** Numbers of detected after instrumentation and post-gutta-percha removal dentin defects.

	After instrumentation			Post-GP removal		
	Craze line	Partial crack	Fracture	Craze line	Partial crack	Fracture
Group 1	46	1	4	47	1	4
Group 2	17	0	3	17	1	3

**Table 2:** Distribution of dentin defects post-gutta-percha removal according to the apical, middle, and coronal portions of the roots.

	Apical	Middle	Coronal
Group 1			
Craze line	18	26	3
Partial crack	1	0	0
Fracture	2	2	0
Total defects	21	28	3
Group 2			
Craze line	7	7	3
Partial crack	1	0	0
Fracture	1	1	1
Total defects	9	8	4
